# Pseudomyogenic hemangioendothelioma secondary to fibrous dysplasia of the left lower extremity in a 14-year-old female: a case report

**DOI:** 10.1186/s12957-016-0955-9

**Published:** 2016-07-28

**Authors:** Conglin Ye, Xiaolong Yu, Jin Zeng, Hucheng Liu, Min Dai

**Affiliations:** 1 Department of Orthopedics, Artificial Joints Engineering and Technology Research Center of Jiangxi Province, The First Affiliated Hospital of Nanchang University, No. 17 Yong Wai Zheng Street, Nanchang, 330006 Jiangxi China; 2 Multidisciplinary Therapy Center of Musculoskeletal Tumor, The First Affiliated Hospital of Nanchang University, Nanchang, 330006 Jiangxi China

**Keywords:** Pseudomyogenic hemangioendothelioma, Fibrous dysplasia, Malignant transformation, Surgery, Imaging, Immunohistochemistry, Pathology

## Abstract

**Background:**

Pseudomyogenic hemangioendothelioma is a rare soft tissue tumor usually found in young adults, predominantly males. Fibrous dysplasia is a common benign bone tumor, which accounts for 5~7 % of all the primary benign bone tumors. However, pseudomyogenic hemangioendothelioma secondary to fibrous dysplasia is extremely rare. To the best of our knowledge, this is the first case of pseudomyogenic hemangioendothelioma secondary to fibrous dysplasia.

**Case presentation:**

This study describes a case of a 14-year-old female who suffered from pseudomyogenic hemangioendothelioma secondary to fibrous dysplasia of the left lower extremity. The patient underwent two operations successively due to pathological fractures in the left femur and tibia in a local hospital. She was diagnosed with fibrous dysplasia according to the postoperative pathological examinations. However, less than 1 year later, she was diagnosed with a recurrence of fibrous dysplasia in her left femur during a follow-up in our hospital. She underwent a curettage and grafting in the left femur. Postoperative pathological examinations demonstrated the diagnosis of fibrous dysplasia. Nevertheless, she presented to our clinic with a chief complaint of pain and swelling in her left tibia and calcaneus 4 months later. The patient underwent fine-needle aspiration in her left tibia. According to the histological and immunohistochemical findings, the diagnosis of pseudomyogenic hemangioendothelioma was confirmed by an expert pathology consultant. Finally, the patient had to undergo an amputation of the left thigh. Postoperative pathological examinations confirmed the diagnosis of pseudomyogenic hemangioendothelioma. Postoperative follow-up at 3 months disclosed no evidence of recurrent disease and no residual side effects from therapy.

**Conclusions:**

Pseudomyogenic hemangioendothelioma is a rare endothelial neoplasm which often mimics myoid and epithelioid tumors morphologically. For the diagnosis, the immunostaining is very important but not decisive and enough. Analysis based on any single factor or incomplete information may easily lead to arbitrary conclusion. Clinical information including age, gender, tumor location, disease course, and recurrence is important for appropriate diagnosis, and full understanding of the tumor is indispensable.

## Background

Pseudomyogenic hemangioendothelioma (PMH), also known as epithelioid sarcoma-like hemangioendothelioma, occurs more frequently in young adult males and usually arises in the extremities, especially on the lower limb, and often involves multiple tissue planes [1–5]. In the current WHO classification of soft tissue tumors, PMH is listed as an intermediate, rarely metastasizing, vascular tumor with peculiar clinical and pathological features [5]. Fibrous dysplasia (FD) is a benign bone lesion that is characterized by the replacement of portions of the normal bone by fibrous connective tissue and poorly formed trabecular bone. Monostotic and polyostotic forms exist. Malignant degeneration is very unusual. The most commonly observed malignant transformations are osteosarcoma, fibrosarcoma, and chondrosarcoma [6]. To the best of our knowledge, this is the first case of PMH secondary to FD. The present study describes the case of a PMH forming secondary to a FD in the left lower limb in a 14-year-old female patient. This study was approved by the ethical review committee of The First Affiliated Hospital of Nanchang University Medical School, and informed consent was obtained from the patient.

## Case presentation

A 14-year-old female attended the Orthopedic Oncology Clinic at The First Affiliated Hospital of Nanchang University (Nanchang, China) for a follow-up. She underwent two operations successively due to pathological fractures in the left femur and tibia in a local hospital about 1 year ago. Then, she was diagnosed with FD according to the postoperative pathological examinations. Recently, she had no obvious symptoms and discomfort. Physical examinations showed nothing remarkable, but a little swelling in her left distal thigh. She had a negative family history of bone tumor. There was no fever or respiratory embarrassment accompanying the swelling. No history of weight loss or exposure to tuberculosis was mentioned.

**Fig. 1:**
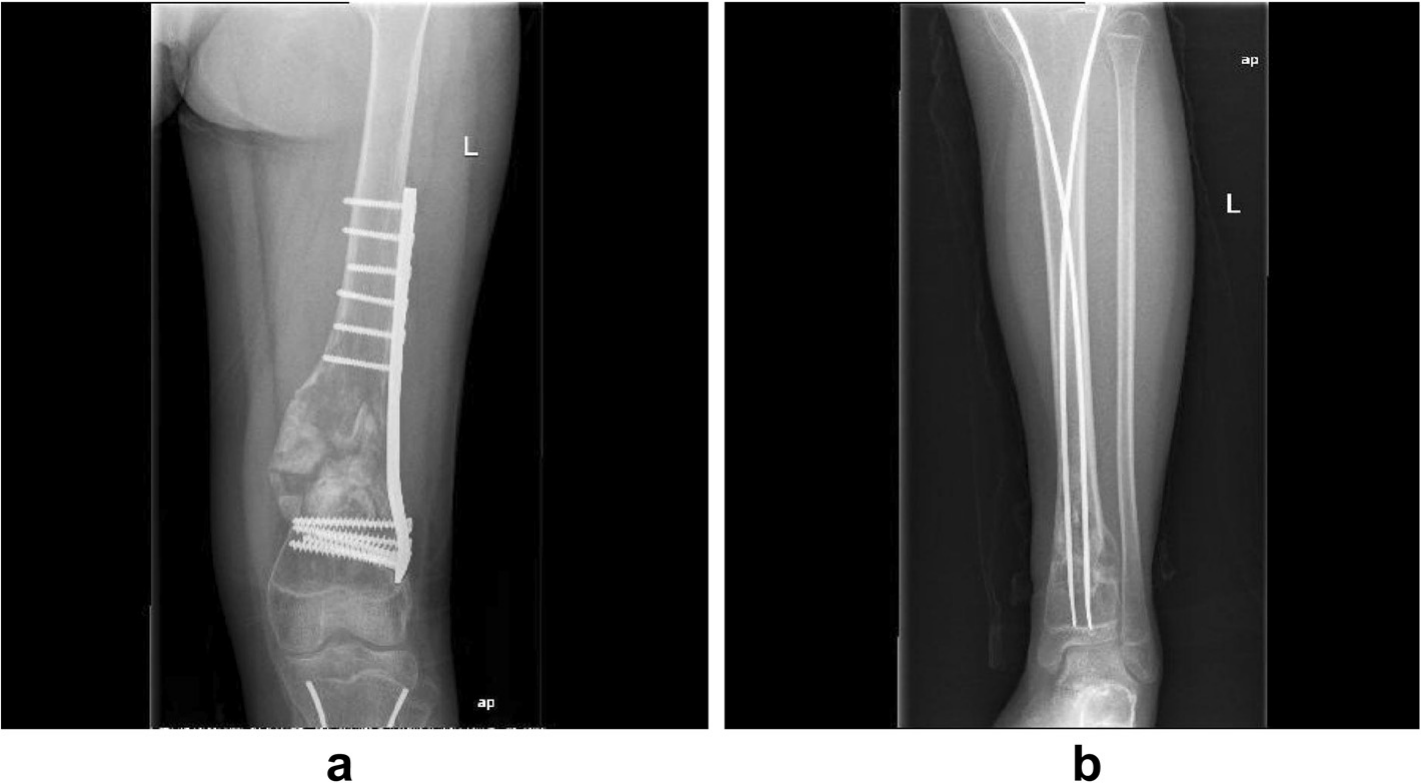
**a** Plain radiographs indicated an expansile osteolytic lesion with marginal sclerosis and a matrix with a ground glass appearance and without periosteal reaction of the left distal femur. **b** Radiographic review showed a prominent osteolytic lesion located in the distal portion of the tibia and calcaneus with cortical destruction

In a further examination, physical examination showed no palpable head, neck, supraclavicular, axillary, or epitrochlear lymph nodes. Inflammatory markers were within the normal limits. Plain radiographs indicated an expansile osteolytic lesion with marginal sclerosis and a matrix with a ground glass appearance and without periosteal reaction of the left distal femur, which was consistent with FD (Fig. 1a). Then, a diagnosis of recurrence of FD in the left femur was made given the medical history of FD, symptoms, and imaging findings.

**Fig. 2:**
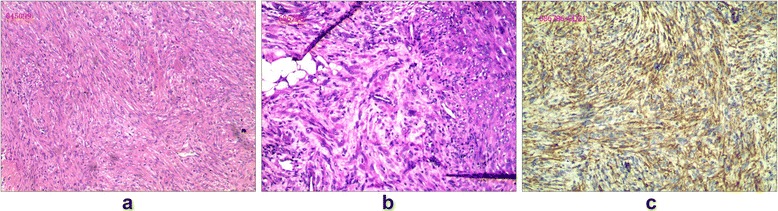
**a** Histologic analysis of hematoxylin and eosin-stained specimens of the left femur showed irregularly shaped spicules of immature bone without osteoblastic rimming and fibrous stroma with few mitotic activities, which consisted in the diagnosis of recurrence of FD of the left femur. **b** The histologic analysis of specimens from the amputation consisted in the diagnosis of PMH secondary to FD of the left lower limb. **c** Immunohistochemical analysis showed positive for CD31

Based on the exclusion of surgical contraindications, professional surgeons, who specialized in treating bone tumors, performed the surgery of curettage and grafting in the left femur. Histologic analysis of hematoxylin and eosin-stained specimens showed irregularly shaped spicules of immature bone without osteoblastic rimming and fibrous stroma with few mitotic activities (Fig. 2a). Immunohistochemical analysis showed positive for CD68 and Vim but negative for bcl-2, CK, CD34, CD99, EMA, SMA, and S100. According to the above findings, the diagnosis of the recurrence of FD of the left femur was finally confirmed.

Nevertheless, she presented to our clinic again with a chief complaint of pain and swelling in her left tibia and calcaneus 4 months later. Radiographic review showed a prominent osteolytic lesion located in the distal portion of the tibia and calcaneus with cortical destruction (Fig. 1b). The patient underwent surgery with the removal of the tumor tissue and reconstruction with allogenous bone graft. According to the histological and immunohistochemical findings, the diagnosis of PMH secondary to FD was confirmed by an expert pathology consultant.

Finally, the patient had to undergo an amputation of the left thigh. The tumor consisted of densely distributed pleomorphic cells, ranging in configuration from small spindled elements to large plump epithelioid variants with prominent pink cytoplasm. Scattered cells with vacuolated cytoplasm were also present. Prominent pleomorphic nuclei contained optically empty centers with peripheral marginalization of the chromatin, in addition to conspicuous nucleoli. Multinucleated cells were also present. Vasoformative elements, such as multi-cellular vascular channels or intracytoplasmic vacuoles, were identified (Fig. 2b). Immunohistochemical analysis showed positive for CK, CD31 (Fig. 2c), but negative for s-100 and CD34. According to the above findings, the diagnosis of PMH secondary to FD of the left lower limb was finally confirmed.

The patient was discharged without any complications 1 week after the amputation. At the time of the 3-month follow-up, the patient reported no pain or discomfort in her left lower extremity. No evidence of recurrence or distal metastasis was noted during the 3 months after surgery. However, it is necessary to perform continuous observations of the patient because of a high rate of recurrence and metastasis.

### Discussion

Pseudomyogenic hemangioendothelioma is a rare soft tissue tumor—distinct from epithelioid hemangioendothelioma [7]—originally described as a “fibroma-like variant of epithelioid sarcoma” [8]; it has been recently concluded that the “epithelioid sarcoma-like hemangioendothelioma” is essentially the same pathological entity [4]. PMH affects predominantly young males. The tumor is multifocal, involving different tissue planes and, although, it shares some histopathologic features with epithelioid sarcoma, it has a different, spindle cell morphology with common positivity for CD31, lack of CD34 reactivity, and intact INI-1 immunoreactivity [2, 9].

By contrast, fibrous dysplasia (FD) is a common tumor-like lesion characterized by solitary or multifocal polyostotic intramedullary lesions [10]. It has a frequency of 2.5 % for all bone lesions and 7 % for benign bone tumors [11]. While any bone can be affected by FD, the most common sites of the disease are the femur, the tibia, the ribs, the skull, the facial bones, the humerus, and the pelvis. Although many bones can be affected at once, FD is not a disease that spreads from one bone to another. Multiple affected bones are often found unilaterally [12].

However, malignant transformation of FD is rare and occurs in less than 1 % of the cases [13, 14]. The most commonly observed malignant transformations are osteosarcoma, fibrosarcoma, and chondrosarcoma [6]. As far as we can see, the current case is the first case reported ever of PMH secondary to FD, which is extremely rare and worthy of special remark.

FD can be divided into two major types: monostotic and polyostotic [15]. According to the largest series, malignant changes seem more likely to occur in polyostotic than in monostotic FD [13, 16]. It is consistent that the current research described a malignant change in a polyostotic FD. Although it is important to recognize malignant transformation as early as possible, it may not be easy as in our case. Suspecting from malignant transformation can be extremely difficult especially in cases with monostotic disease having either subtle symptoms or none. In the reported cases up to date, the special symptoms of the malignant change were mainly pain, swelling, and late appearance of a bony mass [17, 18]. But given the fact that the pain is a nonspecial finding and is the most common complaint followed by pathological fracture in FD [19], great care should be taken when evaluating this symptom. Pain which is rapidly becoming worse over a relatively short period without trauma should be considered alarming [17].

PMH is extremely difficult to diagnose because of no morphological evidence suggestive of endothelial differentiation is present to confirm a radiological pattern of vascular neoplasm (multiple well-limited purely lytic lesions) [2]. The tumor is composed of large spindle cells, arranged in sheets or fascicles. Tumor cells with epithelioid cytomorphology are also often present. In this case, the tumor consists of spindle cells, and round epithelioid cells exist. The cells are large and have abundant eosinophilic cytoplasm, mimicking a myoid tumor or epithelioid carcinoma cells. The tumor cells are plump but show no apparent pleomorphism. The nuclei of the cells have small nucleoli without notable atypia, and the mitotic activity is scarce.

Based on previous reports in other locations, PMH has a more indolent clinical course with a small risk of metastasis [2]. Therefore, complete macroscopic excision is the treatment of choice. Local recurrence must be considered, even with complete, gross surgical resection; close follow-up and adjuvant therapy are warranted.

## Conclusions

In conclusion, we present an extremely rare case of pseudomyogenic hemangioendothelioma secondary to fibrous dysplasia. Pseudomyogenic hemangioendothelioma is a rare endothelial neoplasm which often mimics myoid and epithelioid tumors morphologically. For the diagnosis, the immunostaining is very important but not decisive and enough. Clinical information including age, gender, tumor location, disease course, and recurrence is important for appropriate diagnosis, and full understanding of the tumor is indispensable. Analysis based on any single factor or incomplete information may easily lead to arbitrary conclusion. However, the reports of this tumor are limited. Further studies on more cases are in need for the full understanding and appropriate diagnosis of it. An awareness of the potential for PMH to present in this way is helpful for both radiologists and pathologists when confronted with this rare tumor. With regards to the present case, the patient remained symptom-free 3 months after surgery. Due to the risk of recurrence, and additional risk of metastasis, long-term follow-ups should be continued.

## Abbreviations

FD, fibrous dysplasia; PMH, pseudomyogenic hemangioendothelioma

## References

[1] Billings SD, Folpe AL, Weiss SW (2003). Epithelioid sarcoma-like hemangioendothelioma. Am J Surg Pathol.

[2] Hornick JL, Fletcher CD (2011). Pseudomyogenic hemangioendothelioma: a distinctive, often multicentric tumor with indolent behavior. Am J Surg Pathol.

[3] Cai JN, Peng F, Li LX, Cheng YF, Wang J (2011). Epithelioid sarcoma-like hemangioendothelioma: a clinicopathologic and immunohistochemical study of 3 cases. Zhonghua Bing Li Xue Za Zhi.

[4] Billings SD, Folpe AL, Weiss SW (2011). Epithelioid sarcoma-like hemangioendotheliom (pseudomyogenic hemangioendothelioma). Am J Surg Pathol.

[5] Fletcher CDM, Bridge JA, Hogendoorn PCW, Mertens F (2013). World Health Organization classification of tumors of soft tissue and bone. Pathology and genetics of tumors of soft tissue and bone.

[6] Van Rossem C, Pauwels P, Somville J (2013). Sarcomatous degeneration in fibrous dysplasia of the rib cage. Ann Thorac Surg.

[7] Ma J, Wang L, Mo W, Yang X, Xiao J (2011). Epithelioid hemangioendothelioma of the spine: clinical characters with middle and long-term follow up under surgical treatments. Eur Spine J.

[8] Mirra JM, Kessler S, Bhuta S, Eckardt J (1992). The fibroma-like variant of epithelioid sarcoma. A fibrohistiocytic/myoid cell lesion often confused with benign and malignant spindle cell tumors. Cancer.

[9] Miettinen M, Fanburg-Smith JC, Virolainen M, Shmookler BM, Fetsch JF (1999). Epithelioid sarcoma: an immunohistochemical analysis of 112 classical and variant cases and a discussion of the differential diagnosis. Hum Pathol.

[10] Dorfman HD, Czerniak B. Adamantinoma of long bone: bone tumors. Mosby: St. Louis. 2008. p 441–491. Doi: 10.1186/1746-1596-3-8.

[11] Greenspan A (1992). Orthopedic radiology: a practical approach.

[12] Varghese AI, Harrop CW, Smith WP (2010). Malignant transformation of fibrous dysplasia of the maxilla. Int J Clin Pract.

[13] Schwartz DT, Alpert M (1964). The malignant transformation of fibrous dysplasia. Am J Med Sci.

[14] Yabut SM, Kenan S, Sossons HA, Lewis MM (1988). Malignant transformation of fibrous dysplasia. Clin Orthop.

[15] Kransdorf MJ, Moser RP, Gilkey FW (1990). Fibrous dysplasia. Radiographics.

[16] Horvai A, Unni KK (2006). Premalignant conditions of bone. J Orthop Sci.

[17] Hoshi M, Matsumoto S, Manabe J, Tanizawa T, Shigemitsu T, Izawa N, Takeuchi K, Kawaguchi N (2006). Malignant change secondary to fibrous dysplasia. Int J Clin Oncol.

[18] Xu D, Luan H, Zhan A, Feng W, Sun X, Meng F (1996). Spontaneous malignant transformation of fibrous dysplasia. Chin Med J (Engl).

[19] Saglik Y, Atalar H, Yildiz Y, Basarir K, Erekul S (2007). Management of fibrous dysplasia. A report on 36 cases. Acta Orthop Belg.

